# Investigating *in vivo Mycobacterium avium* subsp. *paratuberculosis* microevolution and mixed strain infections

**DOI:** 10.1128/spectrum.01716-23

**Published:** 2023-08-16

**Authors:** Alexander Byrne, Nathalie Bissonnette, Séverine Ollier, Kapil Tahlan

**Affiliations:** 1 Department of Biology, Memorial University of Newfoundland, St. John’s, Newfoundland and Labrador, Canada; 2 Sherbrooke Research and Development Centre, Agriculture and Agri-Food Canada, Sherbrooke, Quebec, Canada; University of Melbourne, Melbourne, Australia

**Keywords:** Johne’s disease, *Mycobacterium avium *subsp. *paratuberculosis*, whole-genome sequencing, strain typing, molecular epidemiology, microevolution, mixed strain infection, mixed genotype infection

## Abstract

*Mycobacterium avium* subsp. *paratuberculosis* (MAP) causes Johne’s Disease (JD) in ruminants, which is responsible for significant economic loss to the global dairy industry. Mixed strain infection (MSI) refers to the concurrent infection of a susceptible host with genetically distinct strains of a pathogen, whereas within-host changes in an infecting strain leading to genetically distinguishable progeny is called microevolution. The two processes can influence host-pathogen dynamics, disease progression and outcomes, but not much is known about their prevalence and impact on JD. Therefore, we obtained up to 10 MAP isolates each from 14 high-shedding animals and subjected them to whole-genome sequencing. Twelve of the 14 animals examined showed evidence for the presence of MSIs and microevolution, while the genotypes of MAP isolates from the remaining two animals could be attributed solely to microevolution. All MAP isolates that were otherwise isogenic had differences in short sequence repeats (SSRs), of which SSR1 and SSR2 were the most diverse and homoplastic. Variations in SSR1 and SSR2, which are located in *ORF1* and *ORF2*, respectively, affect the genetic reading frame, leading to protein products with altered sequences and computed structures. The ORF1 gene product is predicted to be a MAP surface protein with possible roles in host immune modulation, but nothing could be inferred regarding the function of ORF2. Both genes are conserved in *Mycobacterium avium* complex members, but SSR1-based modulation of *ORF1* reading frames seems to only occur in MAP, which could have potential implications on the infectivity of this pathogen.

**IMPORTANCE:**

Johne’s disease (JD) is a major problem in dairy animals, and concerns have been raised regarding the association of *Mycobacterium avium* subsp. *paratuberculosis* (MAP) with Crohn’s disease in humans. MAP is an extremely slow-growing bacterium with low genome evolutionary rates. Certain short sequence repeats (SSR1 and SSR2) in the MAP chromosome are highly variable and evolve at a faster rate than the rest of the chromosome. In the current study, multiple MAP isolates with genetic variations such as single-nucleotide polymorphisms, and more noticeably, diverse SSRs, could simultaneously infect animals. Variations in SSR1 and SSR2 affect the products of the respective genes containing them. Since multiple MAP isolates can infect the same animal and the possibility that the pathogen undergoes further changes within the host due to unstable SSRs, this could provide a compensative mechanism for an otherwise slow-evolving pathogen to increase phenotypic diversity for overcoming host responses.

## INTRODUCTION

*Mycobacterium avium* subsp. *paratuberculosis* (MAP) causes paratuberculosis or Johne’s disease (JD) in ruminants; a disease which is associated with chronic diarrhea, progressive weight loss, and decreased milk production in cattle (1). The disease is responsible for significant economic losses to the dairy industry due to reduced milk production, increased cattle mortality, and culling of infected animals, as there is currently no effective vaccine or treatment for JD (2). A better understanding of MAP pathogenicity and transmission mechanisms is also important from a public health perspective due to the potential link between MAP and Crohn’s disease in humans, and concerns have been raised regarding the impact of MAP on other chronic diseases and food safety (3 – 6). MAP is transmitted via the fecal-oral route (7, 8) and can survive for extended periods in the environment (9, 10). Therefore, JD control strategies must take a holistic approach to better understand MAP evolution dynamics, the diversity of strains circulating throughout a herd, and the impact of strain diversity on disease progression.

MAP is a member of the *Mycobacterium avium* complex (MAC), a group of slow-growing non-tuberculous mycobacteria, which are opportunistic pathogens of humans and animals (11 – 13). Of the other subspecies of *M. avium*, MAP is most closely related to *M. avium* subsp. *homminissuis*, which typically infects pigs and humans (11, 13 – 15). MAP strains are phylogenetically classified as either sheep-type (S-type/types I and III) or cattle-type (C-type/type II) strains, with the “C-type” also comprising bison-type (B-type) isolates (16 – 18). Classification of strains using the types I, II, and III system is preferred as MAP isolates from “S-type” or “C-type” lineages are not limited to causing infections in sheep and cattle species, respectively (19 – 23). Over the years, additional MAP strain typing methods have been developed, which are based on analyzing differences in the copy numbers or sequences of variable DNA repeats. These include the 8 locus Mycobacterial Interspersed Repetitive Unit-Variable Number Tandem Repeat (MIRU-VNTR) and the 11 locus SSR typing methods, both of which are commonly used in epidemiology and source tracking studies (24, 25). More recently, whole-genome sequencing (WGS) followed by single-nucleotide polymorphism (SNP) analysis has allowed for strain typing at a much higher resolution than previously possible, leading to the deciphering of new information (23, 26 – 28).

Mixed strain infections (MSIs) refer to polyclonal or co-infections involving strains of a single pathogenic species (29 – 32). In some pathogens, an initially infecting strain can undergo within-host evolution to give rise to genetically distinct isolates, which is referred to as microevolution (33 – 36). The presence of genetically distinct variants of a single pathogen species in an infected host, whether it be due to MSI or microevolution, is collectively referred to as mixed genotype infection (MGI) (Fig. S1). Such events are expected to influence the progression and outcome of an infection, along with transmission dynamics, due to differences in the physiological characteristics of isolates (37 – 41). In some pathogens, MGIs lead to treatment complications due to the presence of multiple antibiotic resistance profiles (also referred to as heteroresistance) (42, 43). There is no approved treatment against JD (44), so the evolutionary pressure on MAP is not associated with antibiotic exposure but instead arises from prolonged infection of the host. Most cattle with JD are infected as calves at 6 months of age or younger and often display disease symptoms years later (1, 45). The genome evolution rate of MAP is low, with estimates ranging from 0.1 to 0.5 SNPs/genome/year (23, 27, 46). Therefore, MGIs, and in particular MSIs, could have implications during infections caused by slow-growing pathogens by exposing the host to genetically and phenotypically distinct strains at the same time (1, 47, 48). Blood antigens or other markers that can be used to accurately predict (or diagnose) JD progression are still lacking (47, 49), which could also be a consequence of MGIs. In the case of other diseases, MGIs are known to interfere with the host immune response, possibly due to antigenic differences between the infecting strains (48 – 50).

While MAP MGIs have been reported in dairy animals using multilocus SSR (ML-SSR) (51) and SNP (52, 53) based analysis, a focused study on the topic is lacking (29). To conduct an in-depth investigation into the presence of MAP MGIs in dairy cattle, we used WGS to examine genetic variations in multiple MAP isolates derived from animals shedding high levels of the bacterium. We were able to identify unique ML-SSR and SNP patterns from isolates both within the same animal and between different animals, allowing for the identification of MGIs and, in some cases, providing evidence for either microevolution or MSI events.

## MATERIALS AND METHODS

### Animal selection, sample collection/analysis, and MAP isolation

The Agriculture and Agri-Food Canada Animal Ethics Committee (AAFC-545) provided approval for all animal procedures conducted. Animal selection based on samples (fecal and blood) collected from Canadian dairy herds for diagnosis of JD during the 2013–2017 longitudinal study were performed as previously described (28). The shedding levels of MAP in feces were determined by qPCR using the VetMAX-Gold MAP Detection Kit (Life Technologies, Corp., Austin, TX) employing DNA extraction using the ZR-96 Fecal DNA Kit (Zymo Research Corp., Irvine, CA). Blood was tested using the IDEXX MAP Ab test kit (IDEXX Laboratories, Inc., Westbrook, ME). Up to 10 individual MAP isolates (139 in total) from 14 high-shedding cows (mean age 5.94 years) from four dairy herds were analyzed using previously described procedures and culture conditions (Fig. S2 and S3) (28).

### DNA extraction, genome sequencing, and annotation

Genomic DNA from axenic cultures of MAP isolated colonies was extracted using the Quick-DNA Fecal/Soil Microbe Miniprep Kit (Zymo Research Corp.) using an Omni Bead Ruptor 24 (Omni International Inc., Kennesaw, GA) as described previously (28). DNA shotgun libraries from the 139 isolates were prepared by the Centre d’expertise et de services Génome Québec (Montreal, QC, Canada) and sequenced using 150 bp paired-end reads with Illumina NovaSeq 6000 technology (SP Flowcell). In addition to the 139 field isolates, the reference sequence MAP K-10 was downloaded from the public database (GenBank accession no. NC_002944.2) for use in SNP variant analysis and phylogeny construction.

The sequencing reads were processed using a slightly modified version of the pipeline previously described (28) (Fig. S4). Prokka was run to annotate the assemblies using the sequences of MAP K-10 (type II reference strain) as a trusted annotation file (54). Prokka HMM databases were enhanced with Pfam and TIGRFAM databases to allow for additional accurate protein annotations to be added (55, 56).

### Variant analysis and phylogeny construction

Snippy was used to call variants (including SNPs, insertions, deletions, multi-nucleotide polymorphisms, and complex variations) from the processed FASTQ files for each isolate, using MAP K-10 as a reference sequence (57). The “.consensus.subs.fa” FASTA files generated for each isolate by Snippy were combined into a single FASTA file for further processing using SNP-sites (58). The “.vcf” files produced by Snippy for each isolate that contained information on their genotypes were mapped onto the K-10 genome sequence using Geneious Prime V.2022.1.1 (Biomatters, Inc., USA), allowing for the visualization of variants such as indels and SNPs in the context of the reference genome.

Genetic variant analysis allowed for the construction of SNP-based phylogenies containing all isolates from the 14 animals, as well as those from individual-based animals. Each core SNP tree was created with IQ-TREE which selected the optimal model for tree building (Table S1) using 1,000 bootstraps (59, 60). Tree visualization was performed using ITOL V6 (61).

### MIRU-VNTR and ML-SSR typing

The DNA sequences corresponding to 11 MAP SSR loci (25) were identified *in silico* from the assemblies using Geneious Prime V. 2022.1.1 (Biomatters, Inc., USA). If the number of repeats at the SSR locus was unclear, the individual reads were examined manually to extract the information. MIRU-VNTR repeats were classified using the Tandem Repeats Finder (24, 62). All MIRU-VNTR and ML-SSR data of MAP isolate along with animal ID are listed in Table S2. Farm ID, Animal ID, ML-SSR, and MIRU-VNTR repeat patterns were annotated onto the collective phylogenetic tree using the iTOL visualizing software, allowing for a visual representation of the *in silico* analysis (61). ML-SSR and MIRU-VNTR patterns of isolates from the current study were compared with those found in the MAC-INMV database for classification (63). The discriminatory index (DI) was used to calculate the strain discriminatory power of both the SSR and MIRU-VNTR loci, as previously described (28).

### Prevalence of variable reading frames in SSR loci 1 and 2

In addition to the 139 MAP genomes from the current study, the sequences of 166 other isolates from our collection (28) or publicly available in the NCBI database (*n* = 1,429) (Table S3) were used for analyzing the prevalence of different nucleotide repeats associated with SSR loci 1 and 2 (henceforth referred to as simply SSR1 and SSR2, respectively) (25). The analysis included 1,388 sequences from the NCBI Sequence Read Archive (SRA, as of 14 December 2022), and FASTQ files corresponding to each record were downloaded and compressed using the “gzip” Unix command. Cleaning and assembly of reads from SRA and other NCBI records were performed using fastp and SPAdes v3.15.4, respectively (64 – 66). Sequence selection and extraction of the information at the loci SSR1 and SSR2 were performed using the *in silico*-PCR software package (67). These shortened sequences were examined using Geneious Prime V.2022.1.1 (Biomatters, Inc.), allowing for the compilation of SSR1 and SSR2 repeat numbers and the determination of the reading frame of the gene through examination of the number of variable repeats (Table S3). In some instances, the sequence from SRA records had to be assembled before analysis to obtain unambiguous results.

### Predictive modeling of proteins encoded by genes containing SSR1 and SSR2

Loci listed as proteins WP_010949291.1 and WP_134797017.1 in the K-10 GenBank accession (NC_002944.2), referred to as ORF1 and ORF2 in this publication, contain the SSR1 and SSR2 repeats, respectively. Statistical analysis of both the reading frames and SSR repeat sizes in *ORF1* and *ORF2* was performed using a *χ* ^2^ test of independence using a *P* value threshold of 0.05. The most prevalent repeat sizes for each of the three reading frames within these two loci were identified, and the complete nucleotide sequences of the loci containing these variants were extracted and translated into their amino acid sequences using Geneious Primer V.2022.1.1 (Biomatters, Inc.). BLASTn searches filtered to detect DNA sequences with ≥80% identity and query coverage were used for detecting orthologous genes (68). Representative amino acid sequences of ORF1 and ORF2 from MAP in each reading frame were also queried using BLASTp (68). Conserved domains (CDs; if present) were detected using the NCBI CD tool (69). SignalP 6.0 was used to detect the presence of signal peptides in the predicted proteins (70), whereas ColabFold (71) was used for protein structure prediction using AlphaFold2 along with MMseqs2 with default settings (72, 73). Protein structures were visualized in ChimeraX (74, 75).

## RESULTS AND DISCUSSION

Information on strain dynamics in animals infected with different MAP variants, whether it be due to MSIs or microevolution (collectively referred to as MGIs), are scarce in the current literature (29). Therefore, we obtained multiple MAP isolates from single infected animals for in-depth genomics analysis. As part of a larger ongoing project in 2013–2017, we had access to 2- to 10.5-year-old JD-positive dairy animals from herds located in the provinces of Ontario and Quebec in Canada. These animals (*n* = 555) served as the source of the blood and fecal samples that were collected on a semi-annual basis and were used in the described analysis.

### Host JD status and MAP strain isolation

Serum ELISA and fecal qPCR-based analysis of collected samples were performed as described previously (28). Serum ELISA measures the presence of specific anti-MAP antibodies that indicates whether the host has recognized the pathogen and mounted a humoral response against MAP. The fecal qPCR assay quantifies the amount of MAP DNA present in fecal samples, while the fecal decontamination and culture protocol confirmed the presence of live MAP. To examine the presence of MGIs, 14 high-shedding animals from four farms (three from Quebec and one from Ontario) were selected for further analysis (Fig. S5). The last fecal sample collected from each cow was cultured on solid media confirming the high level of MAP shedding status as described previously (76). Isolation of 10 MAP colonies was performed from each animal (with exception of animal A20 where only 9 colonies could be obtained, Table S2). Each colony was individually grown in liquid media (*n* = 139 axenic cultures) and used for isolating bacterial DNA for whole-genome sequencing using Illumina NovaSeq 6000 technology (28).

### SNP-based phylogenetic analysis

**Fig 1:**
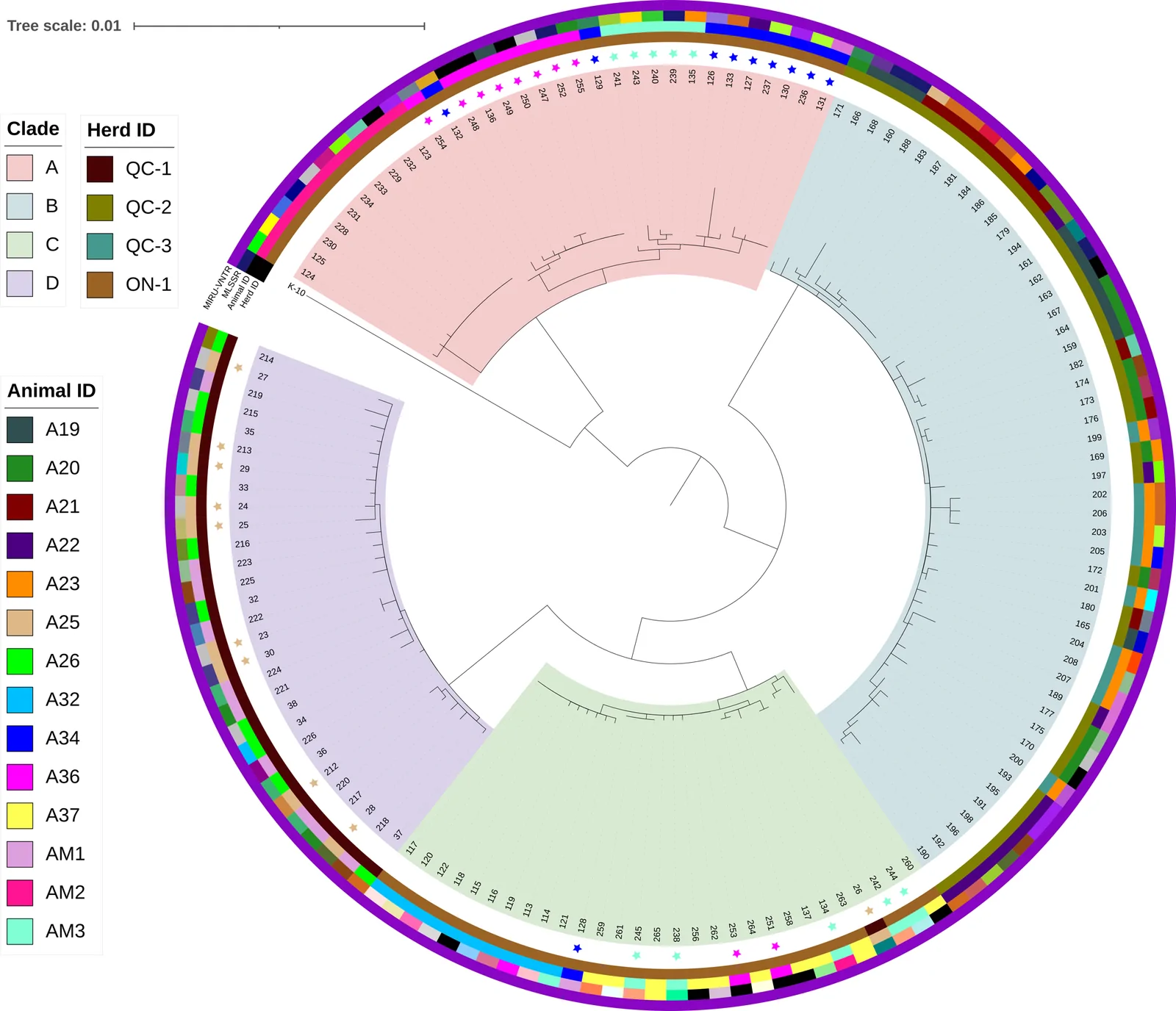
Core SNP-based phylogeny of 139 MAP isolates obtained from the feces of 14 animals examined in the current study. Isolates are distributed among four major clades based on SNP profiles, where each clade has been colored differently as indicated. For each isolate, the concentric circles indicate (from inside to outside): the herd where the animal came from (ID), the animal from which the isolate originated (ID), the ML-SSR pattern, and the INMV MIRU-VNTR type. Colors used for depicting herd and animal IDs are included. ML-SSR patterns labeled in black are unknown, while all other colors represent individual strain types (details are provided in Table S2). All isolates in this study showed the same MIRU-VNTR pattern (INMV 2) as indicated by a single color. The differently colored stars indicate isolates from herds that are distributed among more than one of these four major clades, with the color of the star reflecting the animal the isolate was derived from.

After assembling the sequencing data, the 139 genome sequences were qualified as high quality with an average coverage of 14×, which approached near completeness (99.28%) (Tables S45) (77). Based on genomic markers (23, 28, 78 – 80), all 139 isolates from the current study were classified as type II strains. To identify core genome SNPs for constructing phylogenies for the 139 isolates, MAP K-10, a prototypical type II strain associated with dairy cattle (81, 82), was used as a reference (Fig. 1). Detailed analysis showed that the number of SNP variants detected across all isolates was low (67–101 SNPs), but the diversity increased to 125–158 variants when insertions and deletions were taken into consideration (Table S6).

**Fig 2:**
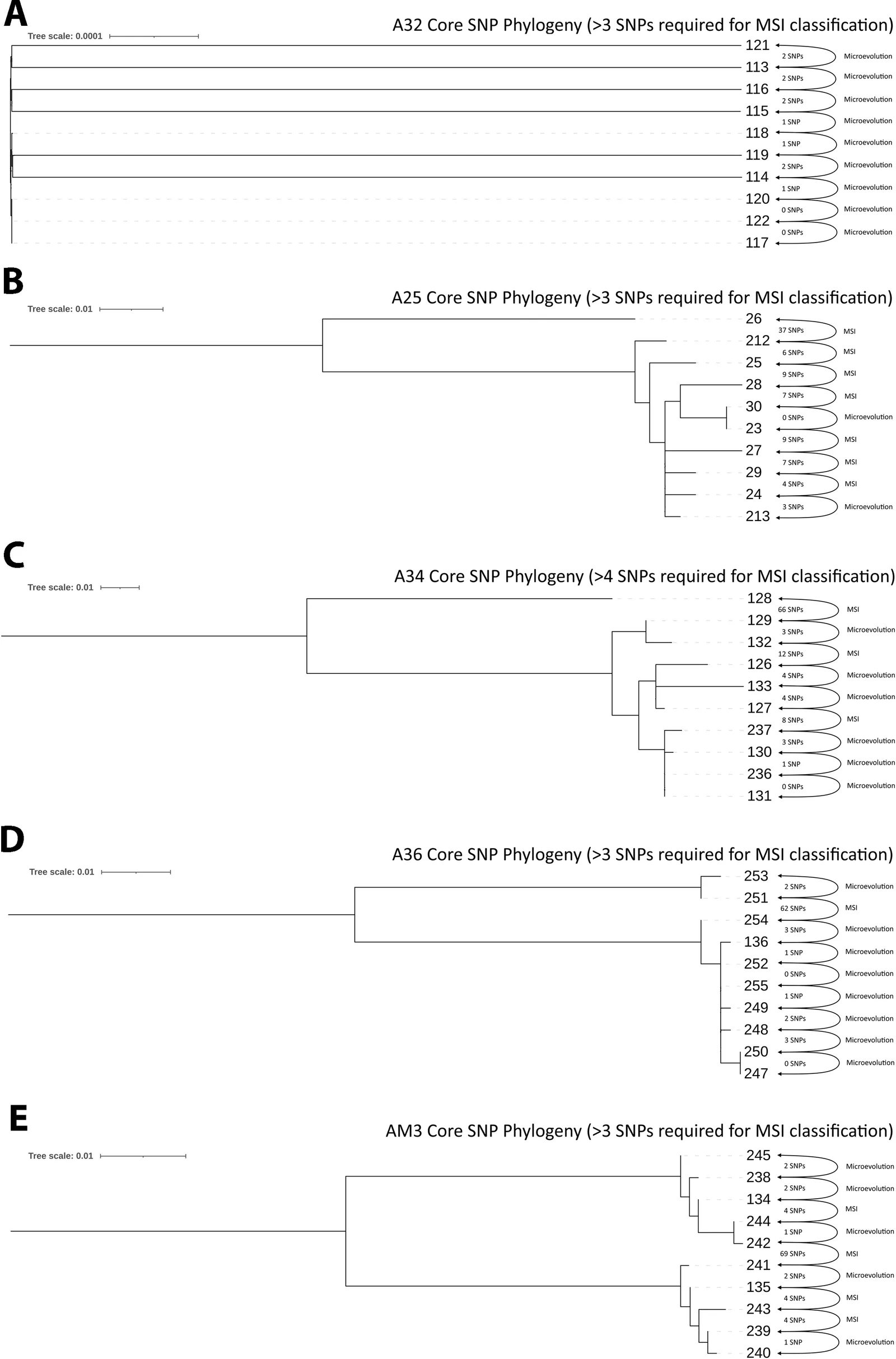
Core SNP phylogenetic trees of select MAP isolates constructed on a per-animal basis. SNPs in all isolates from each animal (*n* = 10, except A20, where *n* = 9) were used for tree construction. The arrows between related MAP pairs derived from the same animal indicate the number of core SNPs that are different between them, and whether this correlates with microevolution or MSI. (**A**) Core SNP phylogeny of MAP isolates from animal A32 as an example where all isolates are closely related, and any SNP differences can be explained on microevolution. (**B–E**) Individual phylogenies of MAP isolates from animals, A25, A34, A36, and AM3, respectively, which were distributed across more than one clade in Fig. 1. Isolates from these four animals had much higher SNP differences, alluding to more instances of MSIs.

Based on core genome phylogenies, MAP isolates from the current study broadly clustered into four clades, where most isolates derived from the same animal were clustering together (Fig. 1). However, this was not the case for four animals (A25, A34, A36, and AM3), which had MAP isolates belonging to different clades (Fig. 1), suggesting that these animals were infected with phylogenetically distinguishable strains and could be classified as having MSIs. The core genomes of MAP isolates derived from the same animal were directly compared with each other to examine this further (Fig. 2; Fig. S5). While pairwise comparisons of isolates from some animals showed that they were closely related (Fig. 2A), MAP isolates from animals A25, A43, A36, and AM3 showed large SNP differences, which confirms the presence of MSIs caused by substantially diverse isolates that could not have arisen by microevolution (Fig. 1). Examination of MAP isolates from animal A25 showed that all isolates cluster together, except for isolate #26 (Fig. 1), which shows an SNP difference of 37 compared to the next closest related strain (Fig. 2B). Similarly, isolate #128 from animal A34 does not cluster with other isolates from this animal (Fig. 1) and differed by 66 SNPs (Fig. 2C). MAP isolates from animal A36 are divided into two clades, with eight clustering together, and the remaining two isolates (#251 and #253) clustering separately (Fig. 1). A pairwise comparison of the closest related isolates between these two clades (#251 and #254) showed a difference of 62 SNPs (Fig. 2D). The 10 isolates from animal AM3 clustered evenly into two different clades (Fig. 1), where isolates #241 and #242 from the two clades differ by 69 SNPs (Fig. 2E). The differences in clustering patterns (Fig. 1) and unique SNP numbers (Fig. 2) of MAP isolates from these four animals strongly suggest instances of MSIs, indicating independent co-infection with different isolates.

### SNP evolution rates and MSIs

The distribution of MAP isolates from the same animal into different clades of the phylogenetic tree provides clear evidence in support of MSI events (Fig. 1), but based on our data, it is possible to examine strain evolution trajectories at a finer level. The genome evolution rate of MAP is very slow, with studies suggesting that changes occur in the range of 0.1–0.5 SNPs/genome/year (23, 27, 46). The animals included in our study were between 4 and 10.5 years of age at the time of sample collection. Working under the assumption that animals were infected soon after birth and using a conservative genome evolution rate of 0.5 SNPs/genome/year, we can calculate the theoretical number of SNPs that would be observed in the sequenced isolates if they evolved from a strain that caused the initial infection (Table S7). By performing SNP analysis on isolates that grouped closely in phylogenetic trees constructed on a per-animal basis (Fig. 2; Fig. S6), we could determine if the number of observed SNPs in each pairwise comparison matched theoretical calculations required to provide support for intra-host microevolution or potential MSI events in the case of discord.

Based on expected differences in MAP SNP frequencies, within-animal comparisons between isolates provided evidence for both microevolution and MSI events in all but 2 of the 14 animals examined (Table 1). MAP isolates from animals A32 and AM2 only showed evidence for potential microevolution and not MSI events, as SNP thresholds for all pairwise comparisons were within the expected range for this to happen, which were three SNPs in both cases (Table S7). As mentioned earlier, four animals showed clear indications of MSI events due to the presence of isolates from distinct clades (Fig. 1 and 2). The evidence provided by examining SNP patterns in pairwise comparisons supports these results and identifies potential MSIs at a higher resolution compared to other genotyping methods (23, 27, 46). Although theoretical SNP calculations suggest clear differentiation between microevolution and MSIs, it cannot be completely ruled out if closely related MAP isolates with SNP differences below the assigned thresholds can infect animals through an MSI event instead of developing through microevolution. In some instances, MAP isolates with identical SNPs had different indels, due to which clonal isolates were not identified in the current study.

**TABLE 1:** Pairwise comparison of SNP differences in isolates from individual animals to provide evidence of microevolution or MSIs

Animal ID	Isolates compared^*a*^	Observed SNP difference	MSI threshold^*b*^	Evidence of MSI or microevolution^*c*^
A19	160 and 168	9	>2	MSI
A19	168 and 166	7	>2	MSI
A19	166 and 161	6	>2	MSI
A19	161 and 165	4	>2	MSI
A19	165 and 162	6	>2	MSI
A19	162 and 163	2	>2	Microevolution
A19	163 and 167	4	>2	MSI
A19	167 and 164	5	>2	MSI
A19	164 and 159	1	>2	Microevolution
A20	170 and 171	6	>4	MSI
A20	171 and 177	2	>4	Microevolution
A20	177 and 175	2	>4	Microevolution
A20	175 and 176	3	>4	Microevolution
A20	176 and 169	0	>4	Microevolution
A20	169 and 172	2	>4	Microevolution
A20	172 and174	4	>4	Microevolution
A20	174 and 173	2	>4	Microevolution
A21	182 and 180	5	>3	MSI
A21	180 and 183	8	>3	MSI
A21	183 and 188	4	>3	MSI
A21	188 and 187	4	>3	MSI
A21	187 and 181	1	>3	Microevolution
A21	181 and 184	1	>3	Microevolution
A21	184 and 186	0	>3	Microevolution
A21	186 and 185	0	>3	Microevolution
A21	185 and 179	0	>3	Microevolution
A22	196 and 190	2	>6	Microevolution
A22	190 and 192	4	>6	Microevolution
A22	192 and 191	7	>6	MSI
A22	191 and 198	2	>6	Microevolution
A22	198 and 193	1	>6	Microevolution
A22	193 and 195	3	>6	Microevolution
A22	195 and 197	7	>6	MSI
A22	197 and 194	4	>6	Microevolution
A22	194 and 189	3	>6	Microevolution
A23	203 and 206	4	>4	Microevolution
A23	206 and 202	4	>4	Microevolution
A23	202 and 208	8	>4	MSI
A23	208 and 199	2	>4	Microevolution
A23	199 and 204	3	>4	Microevolution
A23	204 and 205	4	>4	Microevolution
A23	205 and 201	2	>4	Microevolution
A23	201 and 207	7	>4	MSI
A23	207 and 200	7	>4	MSI
A25	26 and 212	37	>3	MSI
A25	212 and 25	6	>3	MSI
A25	25 and 28	9	>3	MSI
A25	28 and 30	7	>3	MSI
A25	30 and 23	0	>3	Microevolution
A25	23 and 27	9	>3	MSI
A25	27 and 29	7	>3	MSI
A25	29 and 24	4	>3	MSI
A25	24 and 213	3	>3	Microevolution
A26	33 and 215	2	>3	Microevolution
A26	215 and 35	3	>3	Microevolution
A26	35 and 214	5	>3	MSI
A26	214 and 38	7	>3	MSI
A26	38 and 32	6	>3	MSI
A26	32 and 216	6	>3	MSI
A26	216 and 36	4	>3	MSI
A26	36 and 37	2	>3	Microevolution
A26	37 and 34	3	>3	Microevolution
A32	121 and 113	2	>3	Microevolution
A32	113 and 116	2	>3	Microevolution
A32	116 and 115	2	>3	Microevolution
A32	115 and 118	1	>3	Microevolution
A32	118 and 119	1	>3	Microevolution
A32	119 and 114	2	>3	Microevolution
A32	114 and 120	1	>3	Microevolution
A32	120 and 122	0	>3	Microevolution
A32	122 and 117	0	>3	Microevolution
A34	128 and 129	66	>4	MSI
A34	129 and 132	3	>4	Microevolution
A34	132 and 126	12	>4	MSI
A34	126 and 133	4	>4	Microevolution
A34	133 and 127	4	>4	Microevolution
A34	127 and 237	8	>4	MSI
A34	237 and 130	3	>4	Microevolution
A34	130 and 236	1	>4	Microevolution
A34	236 and 131	0	>4	Microevolution
A36	253 and 251	2	>3	Microevolution
A36	251 and 254	62	>3	MSI
A36	254 and 136	3	>3	Microevolution
A36	136 and 252	1	>3	Microevolution
A36	252 and 255	0	>3	Microevolution
A36	255 and 249	1	>3	Microevolution
A36	249 and 248	2	>3	Microevolution
A36	248 and 250	3	>3	Microevolution
A36	250 and 247	0	>3	Microevolution
A37	260 and 265	8	>3	MSI
A37	265 and 256	2	>3	Microevolution
A37	256 and 264	1	>3	Microevolution
A37	264 and 262	0	>3	Microevolution
A37	262 and 263	1	>3	Microevolution
A37	263 and 137	2	>3	Microevolution
A37	137 and 258	3	>3	Microevolution
A37	258 and 261	1	>3	Microevolution
A37	261 and 259	0	>3	Microevolution
AM1	219 and 226	5	>3	MSI
AM1	226 and 220	3	>3	Microevolution
AM1	220 and 218	2	>3	Microevolution
AM1	218 and 217	2	>3	Microevolution
AM1	217 and 221	8	>3	MSI
AM1	221 and 223	6	>3	MSI
AM1	223 and 222	2	>3	Microevolution
AM1	222 and 225	2	>3	Microevolution
AM1	225 and 224	3	>3	Microevolution
AM2	230 and 125	2	>3	Microevolution
AM2	125 and 124	2	>3	Microevolution
AM2	124 and 228	1	>3	Microevolution
AM2	228 and 233	0	>3	Microevolution
AM2	233 and 234	0	>3	Microevolution
AM2	234 and 231	0	>3	Microevolution
AM2	231 and 232	0	>3	Microevolution
AM2	232 and 229	0	>3	Microevolution
AM2	229 and 123	0	>3	Microevolution
AM3	245 and 238	2	>3	Microevolution
AM3	238 and 134	2	>3	Microevolution
AM3	134 and 244	4	>3	MSI
AM3	244 and 242	1	>3	Microevolution
AM3	242 and 241	69	>3	MSI
AM3	241 and 135	2	>3	Microevolution
AM3	135 and 243	4	>3	MSI
AM3	243 and 239	4	>3	MSI
AM3	239 and 240	1	>3	Microevolution

^*a*^ Isolates were selected for pairwise comparisons based on their relatedness in the individual animal phylogenies described in Fig. S4.

^*b*^ SNP differences between isolates were used to calculate thresholds with the assumption that all animals were infected at birth and then using the evolution rates of 0.1 (27) to 0.5 (23) SNPs/genome/year for MAP. To be conservative in our estimation, the calculated values were rounded up to the nearest whole number for determining the maximum number of SNP differences that could be explained based on theoretical evolutionary rates to indicate microevolution. SNP differences between two MAP isolates from the same animal that fell below the calculated threshold suggested potential microevolution, while SNP differences above the threshold were taken to suggest a possible MSI event. Detailed calculations for each of the fourteen animals examined can be found in Table S7.

^*c*^ SNP differences greater than the calculated threshold provide evidence for MSI, while lower numbers are indicative of microevolution.

### *In silico* analysis of repetitive DNA elements

Previous studies have indicated that MAP has a closed pangenome (26, 83, 84), implying that horizontal gene transfer does not significantly contribute to genome evolution in the bacterium (85, 86). The low SNP evolution rate and apparent lack of horizontal gene transfer in MAP make unstable repetitive elements both a driving force for evolution and attractive targets for strain differentiation using molecular methods (87, 88). Both MIRU-VNTR and ML-SSR are significant sources of genetic polymorphisms in mycobacteria and are often used in strain typing (29, 88 – 90). *In silico* analysis of eight MIRU-VNTR loci, specifically MIRUs 292 and X3 along with VNTRs 25, 47, 3, 7, 10, and 32 (24), and 11 SSR loci (25) was performed on the 139 isolates from the current study (Table S2) to determine how they compared with the SNP based phylogenies of the respective isolates.

**Fig 3:**
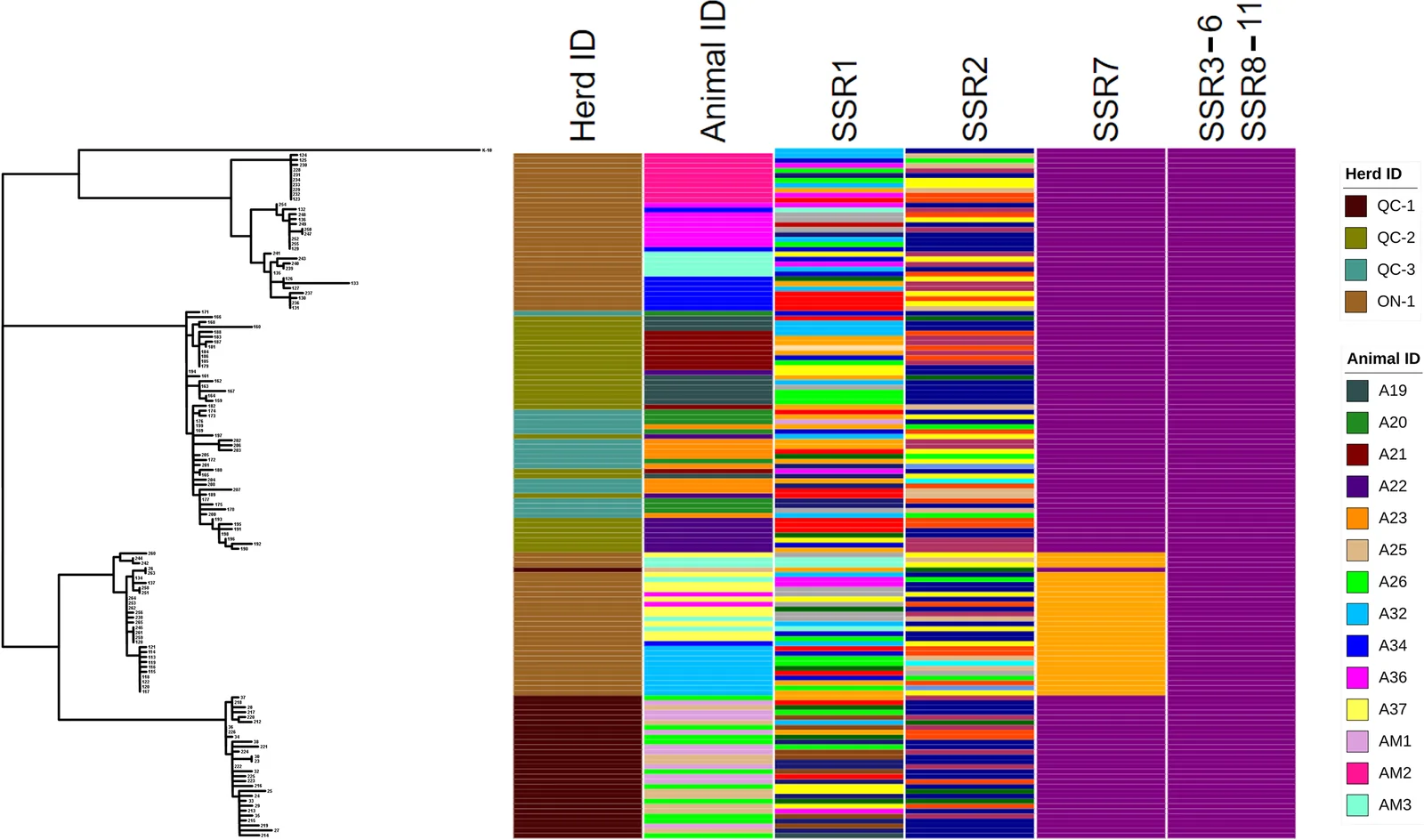
Visual depiction of the SSR diversity observed in MAP isolates from the current study. The phylogenetic tree shown is identical to the one in Fig. 1. Each column represents SSR profiles at a specific locus (1 – 11), where each line indicates the SSR in a MAP isolate, which is color coded according to repeat length. The SSR loci that did not show any diversity (were the same in all isolates: SSR3, 4, 5, 6, 8, 9, 10, and 11) are indicated by the last column on the right for the sake of simplicity.

Of the eight MIRU-VNTR loci analyzed, none showed any variability in the 139 strains examined (DI = 0), due to which only a single INMV-MIRU pattern (63), INMV 2, was detected in our isolates (Table S1). These results agree with previous observations (28), where INMV2 was also the only INMV-MIRU pattern observed in MAP isolates from the same four herds earlier. Complete ML-SSR profiles were also obtained for 127 isolates and were more informative than MIRU-VNTR markers (Fig. 3; Table S8), an observation that has been noted previously (28, 91, 92). The ML-SSR profiles of 12 of the 139 isolates could not be validated and were therefore not used in the analysis. Of the 11 SSR loci examined, SSR1 (*n* = 128), SSR2 (*n* = 138) and SSR7 (*n* = 139) showed variations, whereas the remaining 8 loci had identical repeat patterns in all isolates (Fig. 3; Table S2). SSR1 had the highest discriminatory index value (0.9081), followed by SSR2 (0.8051), then SSR7 (0.3241), which agrees with our previous results (28). For SSR1 and SSR2, 15 and 9 distinct repeat numbers were identified, respectively, whereas SSR7 only showed two different patterns (Table S2).

For the 127 isolates for which unambiguous SSR sizes could be assigned, a comparison of the results to the INMV-SSR database revealed seven matches along with 19 previously unreported ML-SSR patterns (Table S2) (63). Two of these novel patterns (named N/P 5 and N/P 16) were recently described by us (as N/P 12a and N/P 15, respectively) (28), whereas the remaining 17 have not been reported in the literature. One important point to consider is that the MAC-INMV database does not individually classify mononucleotide SSRs greater than 11 nucleotides, but instead groups them together (as “+”). This is normally due to the limitations of conventional DNA sequencing technologies to provide accurate sequences for such repeats (93). In the current study, we used Nova-Seq technology (94), which can provide sequence data for longer repeats leading to new ML-SSR types that would be otherwise categorized as “+.” Four of the INMV patterns identified in our study (ML-SSR 10, ML-SSR 7, ML-SSR 8, and ML-SSR 93) could be classified further due to this increased resolution (Table S8), indicating greater MAP diversity than otherwise suggested by the MAC-INMV database. When accounting for the increased resolution, the total number of unique patterns and the discriminatory index increases from 9 to 71 and 0.7942 to 0.9839, respectively. All 14 animals examined in our study contained distinct MAP isolates with different ML-SSR patterns, with a minimum of 5 and a maximum of 10 patterns found within MAP from each animal (Fig. 1 and 3). Of the 71 ML-SSR patterns found in our study, many were shared by MAP isolates distributed between the four major clades identified based on SNP profiling (Fig. 1).

Examination of certain MAP isolates from the same animal with identical SNPs, indels, and MIRU-VNTR loci, could still be easily differentiated due to variations in SSR1 and SSR2 (Table 2). These findings agree with recent observations made while analyzing single MAP isolates from infected animals, suggesting that ML-SSR typing, like MIRU-VNTR (95), is subject to homoplasy, and do not directly correlate with SNP-based phylogenies (26). Previous studies have shown that microsatellite loci within bacterial genomes, such as the 11 SSR repeats examined here, have a faster rate of evolution compared to the rest of the genome (87, 96). The presence of multiple isolates that are different only at these SSR loci, but are otherwise identical, suggests that the homopolymeric repeats found at SSR1 and SSR2 evolve at a faster rate than the rest of the MAP genome. Therefore, while SSRs might not be good targets for studying phylogenetic lineages due to homoplasy (26), their differential evolutionary rates as compared to the rest of the genome could have significant functional implications, some of which are discussed below.

**TABLE 2:** Isolates from the same animal that differ only in SSR1 and SSR2 repeat sizes

Animal ID	Isolate ID	SSR1 pattern	SSR1 reading frame	SSR2 pattern	SSR2 reading frame
A20	169	17	RF2	12	RF3
A20	176	21	RF3	10	RF1
A21	179	16	RF1	10	RF1
A21	185	18	RF3	11	RF2
A25	23	12	RF3	10	RF1
A25	30	11	RF2	10	RF1
A32	122	15	RF3	12	RF3
A32	117	15	RF3	13	RF1
A36	252	19	RF1	10	RF1
A36	255	18	RF3	10	RF1
A37	262	13	RF1	10	RF1
A37	264	16	RF1	10	RF1
AM2	229	15	RF3	14	RF2
AM2	232	20	RF2	12	RF3

### SSR1 and SSR2 variation and distribution in diverse MAP isolates

Following the observation that MAP homopolymeric repeats associated with SSR1 and SSR2 are highly variable and homoplastic, we set out to examine their diversity and distribution in the genome sequences of all MAP isolates sequenced by our group [*n* = 192 (28); and present study] and those of comparable quality and coverage (*n* = 1429) present in the public SRA and NCBI Nucleotide databases (Table S3). Of the 1621 MAP genomes examined, 182 and 53 sequences for SSR1 and SSR2, respectively, had to be discarded due to ambiguous coverage of the specific genomic loci (Table S3). For the remaining records, the repeat sizes (as nucleotides or nt) at both loci were recorded.

**Fig 4:**
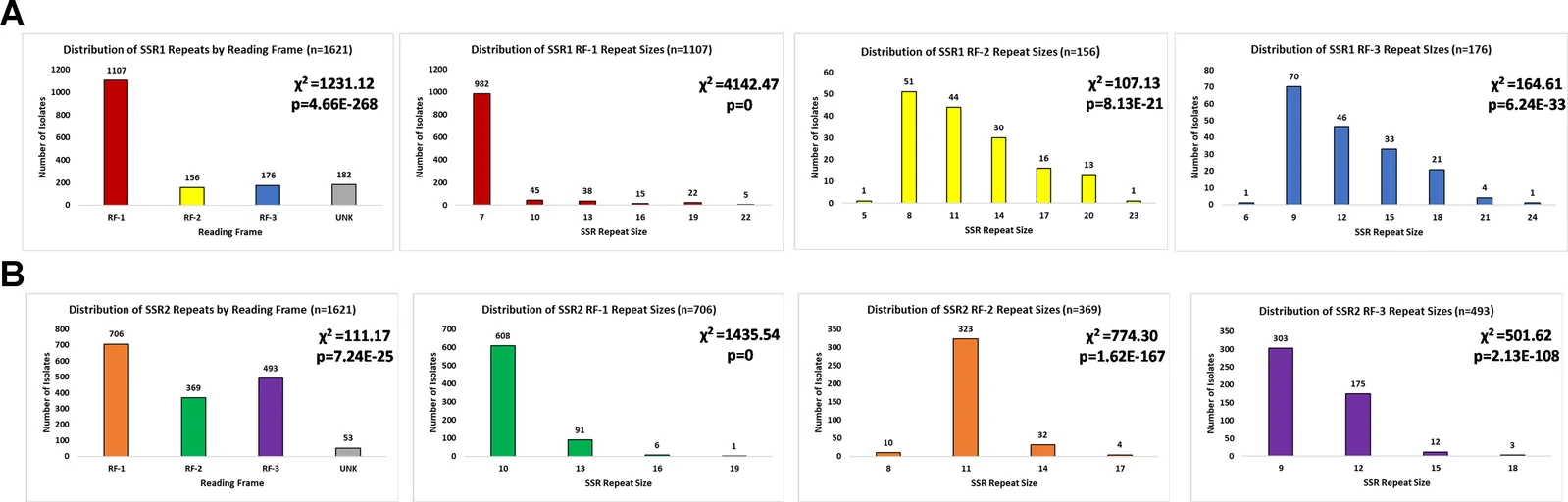
Distribution of (**A**) SSR1 and (**B**) SSR2 repeat sizes in genome sequences of MAP isolates from the current study and those available in public databases. In MAP, SSR1 and SSR2 are situated in *ORF1* and *ORF2*, respectively, and the sequences of the genes in the published MAP K-10 genome sequence were assigned reading frame 1 (RF-1). Based on this, any alteration in the copy number of SSR1 or SSR2 leads to reading frame shifting in the respective *ORF*s (designated as RF-2 and RF-3). (**A and B**) The first panel (from the left) shows the distribution of repeat sizes among the three possible reading frames for each *ORF*, whereas the following panels show the distribution of repeat sizes within RF-1, RF-2, and RF-3 for the respective *ORF*s. The number of genome sequences used in each analysis are included (*N*) along with the *χ*2 test of independence and *P* values obtained.

**Fig 5:**
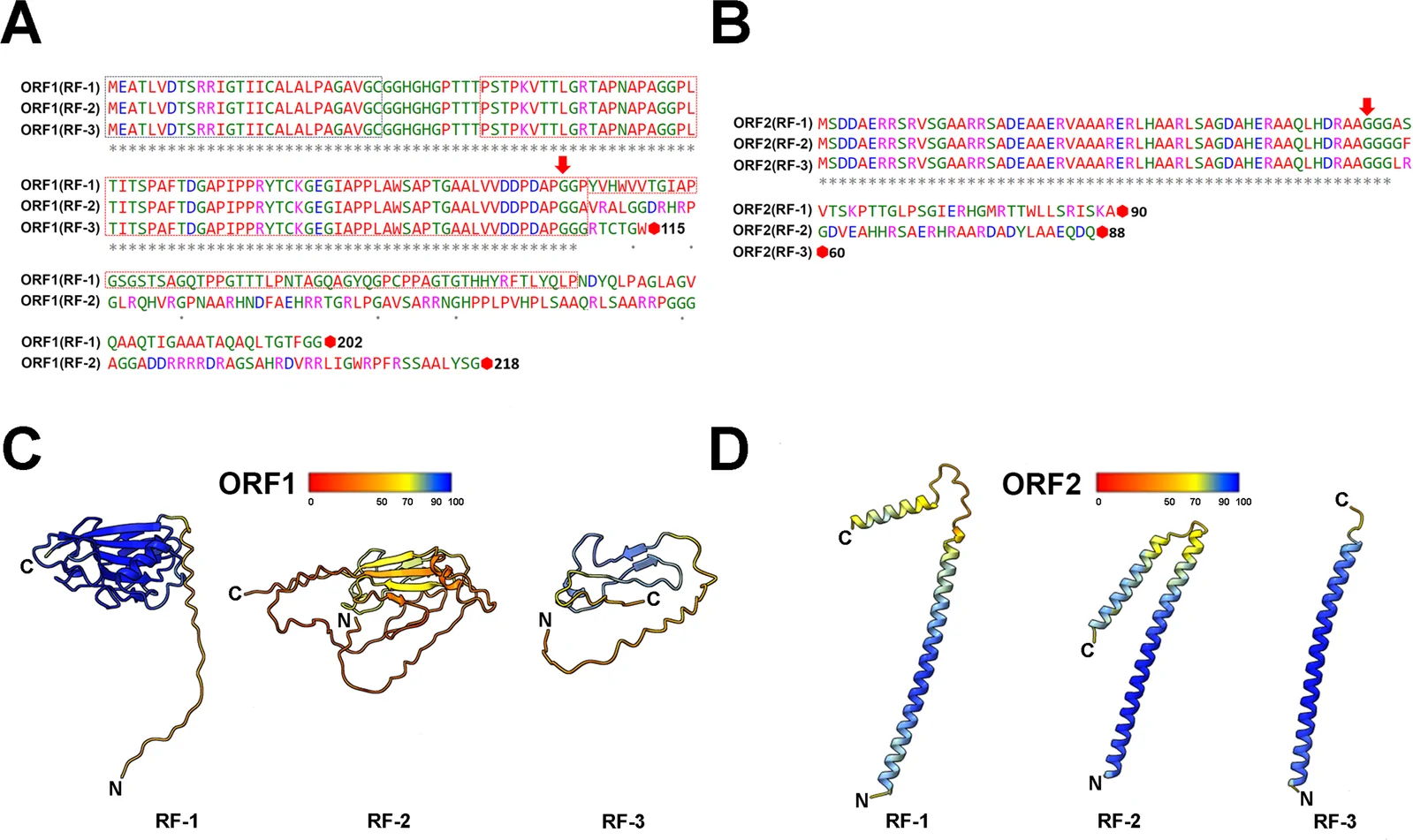
Predicted impacts of reading frame shifts caused by SSR1 and SSR2 variations on the putative ORF1 and ORF2 proteins, respectively. Protein alignments of (**A**) ORF1 and (**B**) ORF2 show products from each reading frame (RF-1 RF-2, and RF-3), respectively. The most common SSR repeat sizes for each reading frame (as indicated in Fig. 4) were used to obtain the amino acid sequences of the proteins shown. (**A and B**) The red arrows indicate the glycine repeat encoded by the variable SSRs. For each ORF, amino acids that are identical across all three sequences are indicated by (*) while those conserved in two of the three variants in each alignment are indicated by (●). Stop codons (red hexagons) and the number of residues comprising each protein are also indicated. (**A**) The gray box indicates the Sec II signal peptide sequence in the three ORF1 variants, while the red box represents the phosphatidylethanolamine-binding (PEBP) domain identified in each sequence. Predicted protein structures of (**C**) ORF1 and (**D**) ORF2 were obtained using Alphafold2. (**C and D**) The computed structures of protein variants corresponding to the three reading frames (RF-1, RF-2, and RF-3) are shown, and their N and C termini are indicated. Coloration is based on the predicted local distance difference test (pLDDT) score for confidence in the structures, where blue indicates high confidence and red regions low, as indicated by the scale.

SSR1 and SSR2 are located in open reading frames that we have designated as *ORF1* (K-10 locus tag MAP_RS08325) and *ORF2* (K-10 locus tag MAP_RS23340), respectively, in the annotated MAP K-10 genome (81, 82). The DNA sequences of *ORF1* and *ORF2* in the reported MAP K-10 genome sequence contain 19 and 10 repeats for SSR1 and SSR2, respectively, and were assigned to be in Reading Frame-1 by us (RF-1, Table S3). Based on this assignment, we classified different SSR1 and SSR2 repeats into three reading frames (RF-1, RF-2, or RF-3) depending on how they affected the predicted amino acid sequence of the cognate gene product (Fig. 4 and 5; Table S9). SSR1 showed a total of 1107, 156, and 182 records which were categorized as RF-1, RF-2, and RF-3, respectively (Fig. 4A). In comparison, SSR2 had a total of 706 records classified as RF-1, 369 as RF-2, and 493 as RF-3 (Fig. 4B). Therefore, it appears that there is some bias in SSR length selection at both loci to maintain the respective ORFs in RF-1, which seems to be more prominent for ORF1 (Fig. 4B through D and 5B through D; Table S3). The prevalence of the most common mononucleotide repeat lengths for SSR1 and SSR2 were examined for each reading frame to determine if there were any preferences (Fig. 4A and 5A). The most common repeat sizes for the reading frames in SSR1 were 7 nt (RF-1, 88.71% of repeats), 8 nt (RF-2, 32.69% of repeats), and 9 nt (RF-3, 39.77% of repeats) (Fig. 4A), while the most common repeat sizes for the reading frames in SSR2 were 10 nt (RF-1, 86.12% of repeats), 11 nt (RF-2, 87.53% of repeats), and 9 nt (RF-3, 61.46%) (Fig. 4B).

The observed distribution of *ORF1* and *ORF2* reading frames and repeat numbers for SSR1 and SSR2 were found to be statistically significant according to a *χ* ^2^ goodness of fit test, suggesting that they were not distributed equally among the population (Fig. 4 and 5). Although there seems to be some preference for SSR1 and SSR2 lengths, variations result in the shifting of the entire reading frame of the respective genes containing them. Several bacterial and fungal species have mechanisms that allow for phenotypic switching (97, 98), enabling them to reversibly alter select phenotypes. One such mechanism used for phenotypic switching is slipped strand mispairing (SSM) during DNA replication, which is especially prevalent in SSR regions, making them prone to insertions and deletions (96, 99, 100). Therefore, the presence of hypervariable homoplastic repeats in *ORF1* and *ORF2* in the otherwise slowly evolving MAP genome suggests that SSR1 and SSR2 provide a mechanism to promote genetic changes to alter the functions of the respective gene products.

### Predicted influence of SSR1 and SSR2 on genes containing them in MAP

The predicted ORF1 product (WP_010949291.1) from MAP K-10 (RF-1) comprises 207 amino acids, where the contained SSR1 encodes for a series of glycines beginning at residue 107 (Fig. 5A). In addition, all three versions of ORF1 (RF-1, RF-2, and RF-3) contain Sec-dependent secretion signals at their N-termini (70, 101), with signatures that suggest signal peptidase II (SPII) dependent processing and membrane attachment (102 – 104) (Fig. S6). Therefore, all the ORF1 variants are predicted to be surface-exposed proteins in MAP and are homologous (80%–100% identity and coverage) to phosphatidylethanolamine-binding (PEB) domain (COG1881) containing mycobacterial hypothetical proteins listed as YbhB/YbcL family Raf kinase inhibitor-like proteins. Further examination of *ORF1* confirmed that the gene is only found in mycobacteria, specifically in members of MAC, including *Mycobacterium avium* subsp. *lepraemurium,* which has also been classified as a member of the complex recently (105, 106) (Table S10). In addition, *ORF1* orthologs from all other MAC members match the RF-1 homolog, with no evidence of the variable region observed in MAP (Fig. 5A). Members from the PEB protein (PEBP) superfamily (also referred to as Raf-kinase inhibitor proteins, RKIP) are found in diverse organisms where they serve a variety of roles, including regulating Raf/MAP kinase and NF-κB signaling pathways in mammals (107 – 109), which in turn control immunity, stress responses, apoptosis and differentiation. In bacteria, PEBP/RKIP proteins from the YbhB/YbcL family have proposed roles in the phosphorylation-based regulation of signaling pathways (110), and YbcL has been noted to contribute to immune modulation in uropathogenic *Escherichia coli* (111). While many PEBP/RKIP proteins only have intracellular functions, some PEBP proteins, such as mammal protein PEBP4 (and its various homologs), can also be secreted (112, 113).

*In silico* examination of the three *ORF1* variants expected to be produced due to reading frame shifts based on different SSR1 lengths indicate that the first 106 amino acid residues, including the Sec-signal sequence, are conserved in all of them (Fig. 5A). The SSR1 sequence encodes a series of glycine residues (starting at residue number 107), the number of which changes depending on the length of the repeat present. Different SSR1 lengths alter the reading frame, leading to the production of proteins with different sizes and amino acid sequences in the region following the glycine repeat (Fig. 5A). In addition, the PEBP domain of all three ORF1 variants begins at residue 39 and varies in size depending on the protein product. Differences were also observed in the predicted structures of the three ORF1 variants obtained using AlphaFold2 (Fig. 5C), which was implemented using Colabfold (71). A high-confidence predicted structure for the C-terminal region of ORF1 could be obtained for RF-1, which was not the case for RF-2 and RF-3. This drop in protein structure confidence suggests that the latter two variants adopt structures that are significantly different and cannot be confidently predicted based on currently available templates and algorithms (114 – 116). Due to evidence suggesting the export of ORF1 through the Sec/SPII pathway, this would allow for the expression of the PEBP domain and subsequent region on the extracellular surface, where the observed variability between reading frames may have an impact on antigenic variation or other host-pathogen interactions (117, 118).

In comparison, the predicted ORF2 product from MAP K-10 (WP_134797017.1) comprises 91 amino acids, where SSR2 encodes a series of glycines starting at residue 56. All ORF2 variants arising from the three different reading frames are predicted to be cytoplasmic, and hypothetical proteins similar to ORF2 (with 80%–100% identity and coverage) are only present in MAC members. In addition, ORF2 ortholog from *M. avium* subsp. *avium*, *M. avium* subsp. *hominissuis* and *M. avium* subsp. *lepraemurium* sequences also contain the SSR2 homopolymer sequence. Only a single *M. avium* subsp. *lepraemurium ORF2* sequence (CP021238.1) where SSR2 comprised 9 nt, was identified during our homology searches (Table S10). In the reported *M. avium* subsp. *avium* and *M. avium* subsp. *hominissuis* sequences, SSR2 ranges from 9 to 11 nt, suggesting the production of protein variants in MAP, *M. avium* subsp. *avium* and *M. avium* subsp. *hominissuis* due to frame shifting (Table S10). As with ORF1, the impact of reading frame shifts caused by SSR variation in MAP ORF2 products was also examined. The first 55 residues preceding the glycine repeat are identical in all three ORF2 variants, which otherwise differ in their C-terminal sequences and overall sizes (Fig. 5B). The impact of these variations is highlighted in the generated high-confidence AlphaFold2 structures, suggesting that ORF2 (RF-1) and RF-2 comprise two alpha helices, while RF-3 only contains one (Fig. 5D), which in theory should lead to major alterations in cognate protein activity.

### Conclusions and perspectives

By analyzing the genome sequences of MAP isolates from the same animal, we were able to identify MGIs and further distinguish them into those resulting from microevolution versus MSIs. MGIs could also be classified into different categories based on the genetic relatedness of MAP isolates, ranging from those caused by MAP with diverse SNP-based phylogenies to ones where the isolates were more closely related to each other. It is important to note that while distinction between microevolution and MSI events can be likely determined using SNP-based calculations, the proposed stable nature of the MAP genome warrants further investigations on the *in vivo* contributions of the two processes. From our results, we noted that SSRs allowed for enhanced discrimination between MAP isolates when complemented with SNP analysis, even though the former are homoplastic, and both methods were more discriminatory than MIRU-VNTR. Maximum diversity was observed in the case of SSR1 and SSR2, and in some instances, they were the only sequences that differed between otherwise isogenic MAP isolates (Fig. 2; Table 2). It was also noted that all *ORF1* sequences from other MAC subspecies corresponded to RF-1 and almost all contained the seven-nucleotide “GGCGGGG” sequence instead of the “G” homopolymer present in MAP. *M. avium* subsp. *lepraemurium* had the most dissimilar sequence, which corresponded to “AGCGGGG.” Our analysis showed that the most common SSR1 repeat size in MAP was 7 (Fig. 3; Table S3), resulting in a protein sequence that matches the predicted *ORF1* from *M. avium* subsp. *avium*, *M. avium* subsp. *hominissuis*, and *M. avium* subsp. *lepraemurium* (Table S10). It is currently accepted that MAP evolved from *M. avium* subsp. *hominissuis*, which is somewhat supported based on comparisons of *ORF1* and *ORF2* sequences (11, 13, 119). As mentioned earlier, the *M. avium* subsp. *avium* and *M. avium* subsp. *hominissuis* orthologous *ORF1* contains a “GGCGGGG” sequence, raising the possibility that a C→G transversion in the sequence of an ancestral MAC member led to the establishment of the unstable SSR1 mononucleotide homopolymer found in MAP (120, 121). This in turn resulted in the variety of repeat sizes reported for SSR1 in MAP, while also affecting the reading frame of *ORF1* (Table S10). In the case of *ORF2*, the *M. avium* subsp. *avium*, *M. avium* subsp. *hominissuis* and *M. avium* subsp. *lepraemurium* orthologs also contain the SSR2 homopolymer, where the *M. avium* subsp. *avium* and *M. avium* subsp. *hominissuis* sequences exhibit variations similar to those observed in MAP. As with MAP, this suggests that *M. avium* subsp. *avium* and *M. avium* subsp. *hominissuis* may have a similar capacity to produce different gene products using alternate reading frames.

In general, variations caused in the amino acid sequences and corresponding structures of MAP proteins due to the presence of variable SSRs in cognate genes have not received much attention. The ORF1 and ORF2 variants predicted in the current study reinforce the importance of variable genetic elements in affecting protein products, beyond just being used as molecular targets in strain typing studies. While the described *in silico* protein analysis reveals novel information, not much is known about the functions of ORF1 and ORF2, beyond that ORF1 is a predicted surface-exposed protein possibly involved in modulating host responses based on its homology to other known proteins (110, 122). It can be hypothesized that changes in the cognate *ORF1* product caused by SSR-associated frame shifting could potentially affect the interaction of MAP with the host, a point that we intend to test in future studies. It is currently not known how these SSRs change or evolve in infecting MAP isolates, but regardless of their origin, MAP with different SSRs in the same animal can be categorized as causing MGIs for now since they are technically not isogenic.

There have been some studies on coinfections involving unrelated species of pathogens in humans and animals (123 – 126), and viral MGIs have also been documented (127 – 130). In comparison, not much is known about the implications of bacterial MGIs, but such phenomena have been modeled or studied using other unicellular eukaryotic pathogens (131). For example, MSIs in mice caused by either *Plasmodium chabaudi* (132) or *Trypanosoma brucei* (133), the causative agents of rodent malaria and human African sleeping sickness, respectively, can lead to mutual suppression or decreased disease severity during the acute phase of infection. In comparison, during the chronic phase of infection, competition between different pathogen strains is low and MSIs are harder to control by the host immune system as compared to clonal infections caused by a single strain (130). Since JD is characterized by having a long subclinical phase of infection with unpredictable disease progress, it remains to be determined if an interplay by different MAP genotypes could have similar effects on either pathogen clearance or disease progression to full-blown clinical JD. The fact that MAP can survive in the environment, multiple strains can infect animals, and the possibility that the pathogen can further evolve within the host due to unstable genetic elements, could have major implications on the spectrum of clinical outcomes and our ability to control JD.

## Data Availability

The 139 MAP genome sequences obtained as part of this study are associated with BioProject PRJNA925907 and have been deposited in the NCBI Sequence Read Archive (SRA) repository under the accession numbers: SRR23179790-SRR23179792, SRR23179800, SRR23179819, SRR23179829, SRR23179835, SRR23179843, SRR23179850, SRR23179851, SRR23179853, SRR23179854, SRR23179855, SRR23179856, and SRR24326188-SRR24326312. References and accession numbers for all other publicly available data sets used are included in Table S3.

## SUPPLEMENTAL MATERIAL

Supplemental Figures spectrum.01716-23-s0001.pdf Figures S1-S7.

Supplemental Tables spectrum.01716-23-s0002.xlsx Tables S1-S10.

ASM does not own the copyrights to Supplemental Material that may be linked to, or accessed through, an article. The authors have granted ASM a non-exclusive, world-wide license to publish the Supplemental Material files. Please contact the corresponding author directly for reuse.
