# Supplementary material for: Investigating in vivo Mycobacterium avium subsp. paratuberculosis microevolution and mixed strain infections
Source: Microbiol Spectr. 2023 Aug 16;11(5):e01716-23. doi: 10.1128/spectrum.01716-23 (PMC10581078; doi:10.1128/spectrum.01716-23)
Supplement: Supplemental Figures — Figures S1-S7. [file spectrum.01716-23-s0001.pdf]

Supplementary Figure S1: Schematic distinguishing between mixed strain infections (MSI) and microevolution. During MSI, different strains infect a single host at the same time, as represented in the black rectangle on left. In microevolution, a single strain infects a host and undergoes within host evolution, resulting in genetically related lineages (represented in the black rectangle on right). Both MSI and microevolution can be categorized under the umbrella term of mixed genotype infection (MGI).

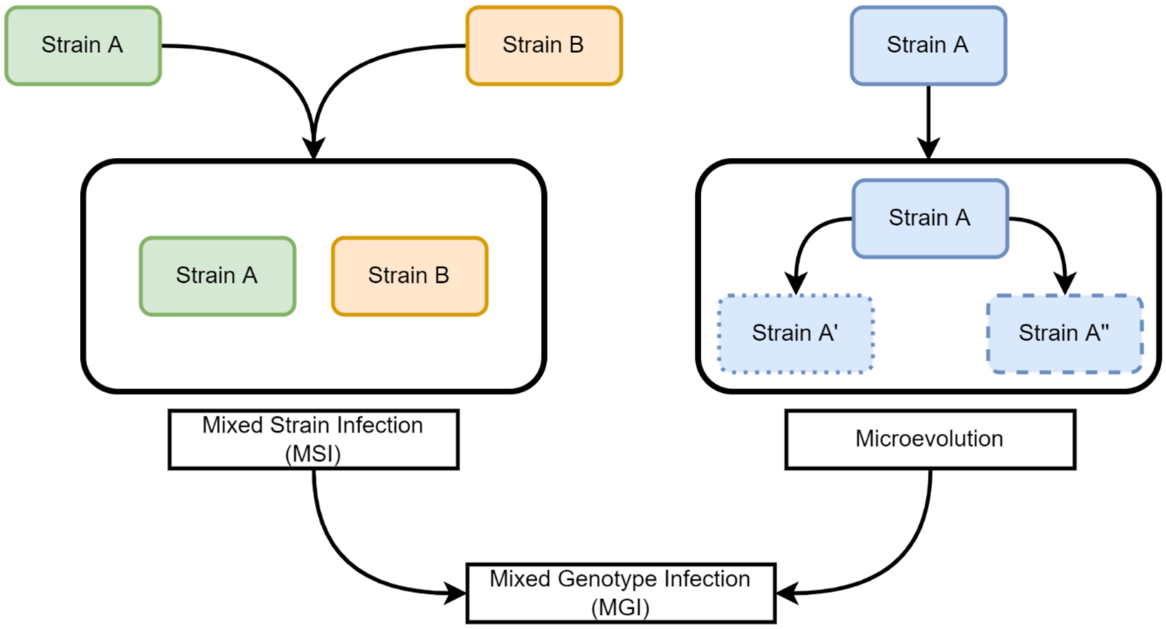

Supplementary Figure S2: Procedure followed to decontaminate fecal samples and prior to culturing MAP according to Byrne et al., 2023.

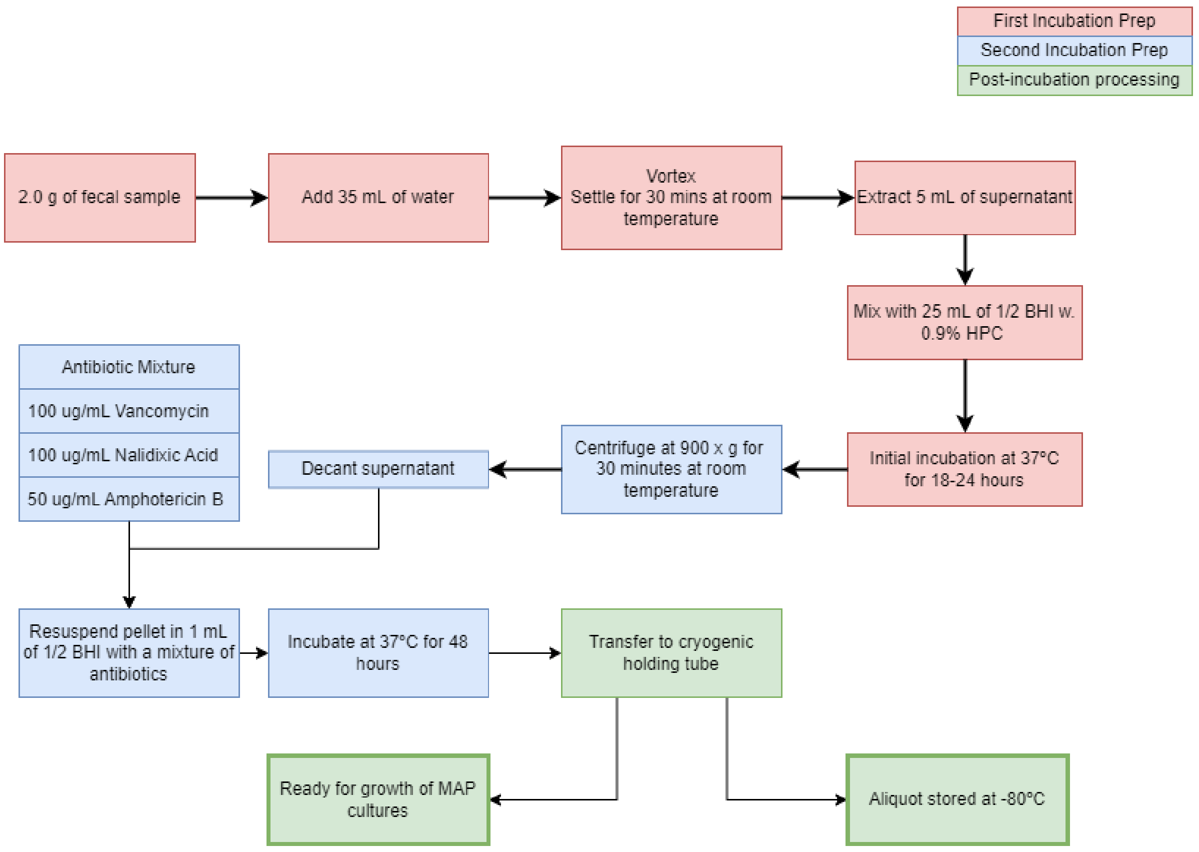

Supplementary Figure S3: Schematic of the procedure used to culture MAP isolates from high shedding animals according to Byrne et al., 2023.

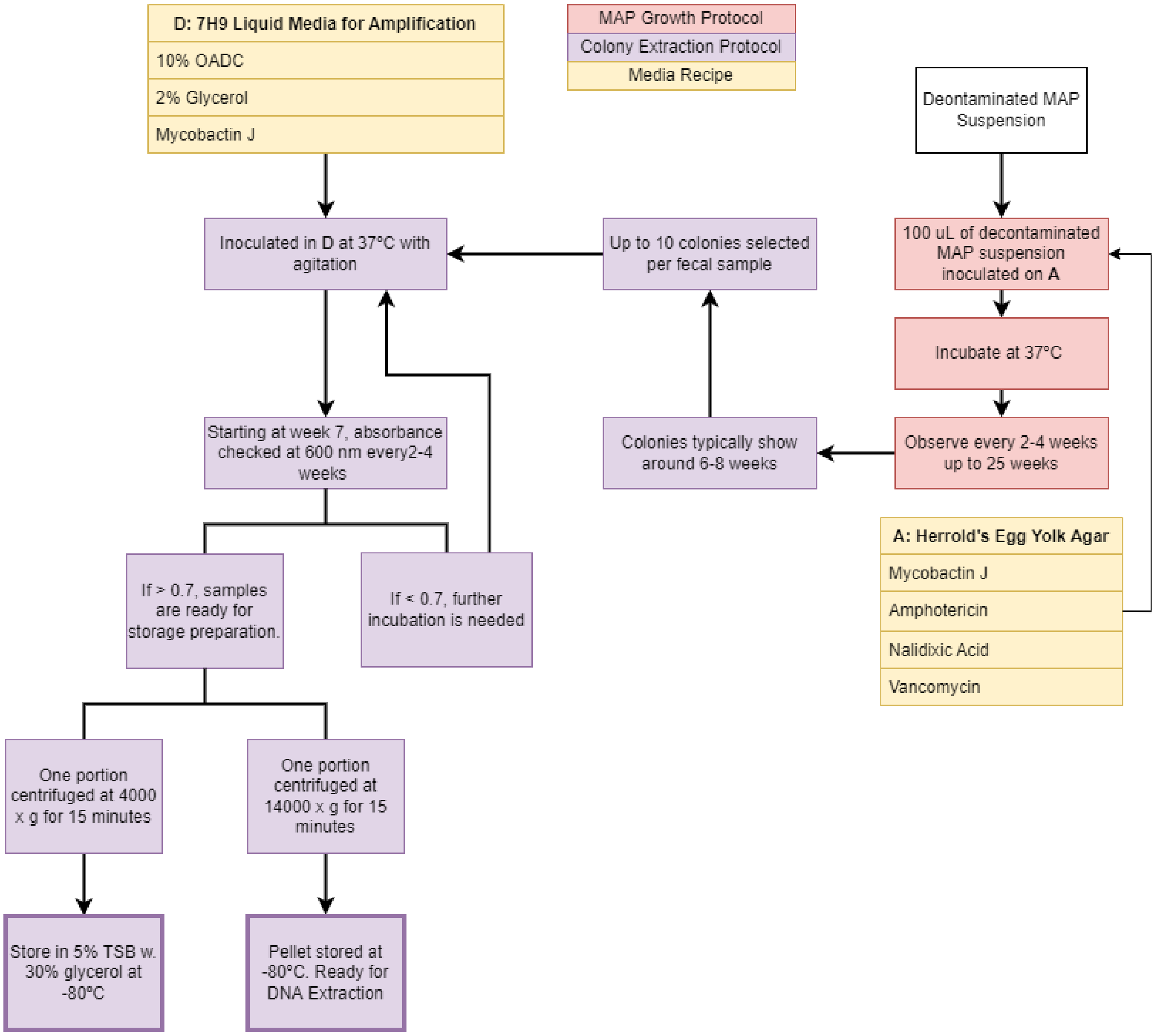

Supplementary Figure S4: Pipeline of bioinformatic tools (diamonds) and files (rectangles) used to analyze NovaSeq 6000 whole genome sequencing reads. Additional details are described in Byrne et al., 2023.

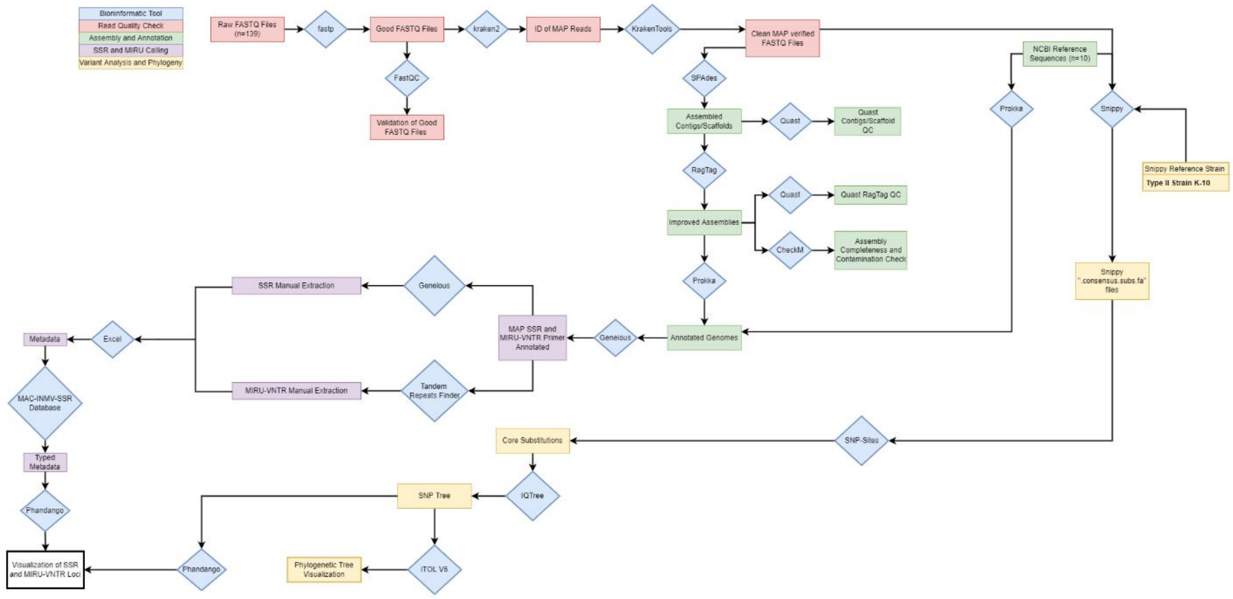

Supplementary Figure S5: Graphical plots showing the relationship between the ages of all 14 animals included in the current study, levels of MAP shed in their feces (qPCR cycle threshold, or C_t value) and blood ELISA (sample/positive, or %S/P) results. All animals were classified as high shedders ($C_t < 27$) and the arrows on each graph represent the timepoints at which fecal samples were collected for MAP culture from each animal. The grey line with circles (●) represents the %S/P values of the serum ELISA, whereas the black line with crosses (x) represents the C_t values from fecal qPCR analysis. The dashed lines along the bottom of each graph represents the ELISA threshold (45% S/P) for a cow to be suspected of being MAP infected positive (light gray), and the ELISA threshold (55% S/P) for a cow to be considered JD positive, as recommended by the manufacturers of the assay kit used for analysis.

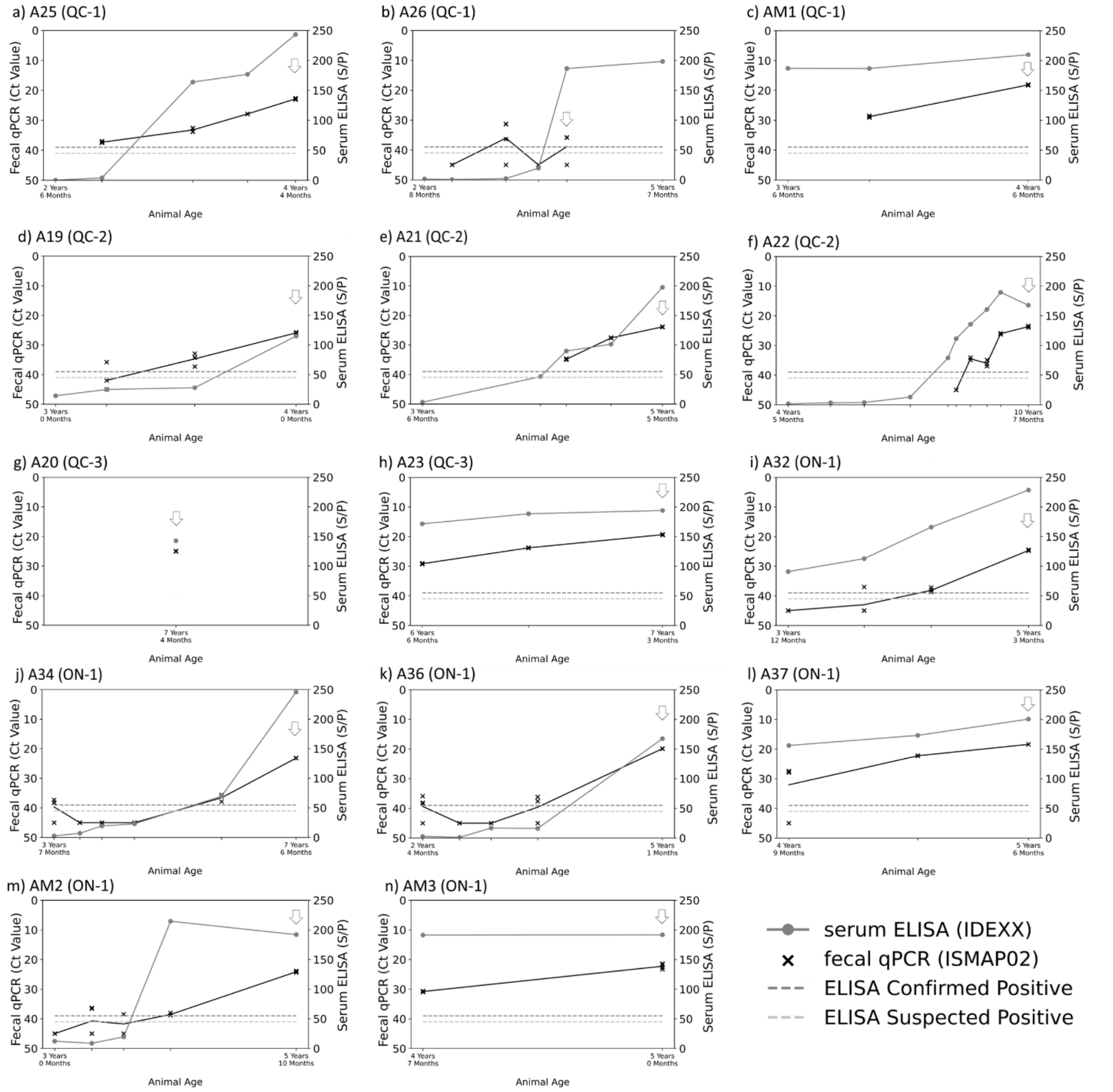

Supplementary Figure S6: Phylogenetic trees of MAP isolates constructed on a per-animal basis. The SNPs for all isolates (n=10) from each animal in the current study (except A20, where n=9) were used for tree construction using IQ-TREE and visualization using iTOL V6. Phylogenies of MAP isolates from animals A19 **(a)**, A20 **(b)**, A21 **(c)**, A22 **(d)** Phylogeny for A23 **(e)**, A26 **(f)**, A37 **(g)**, AM1 **(h)** and AM2 **(i)** are shown. In each case the number of SNPs required to classify isolates arising from MSIs are indicated.

(a) A19 Core SNP Phylogeny (>2 SNPs required for MSI classification)

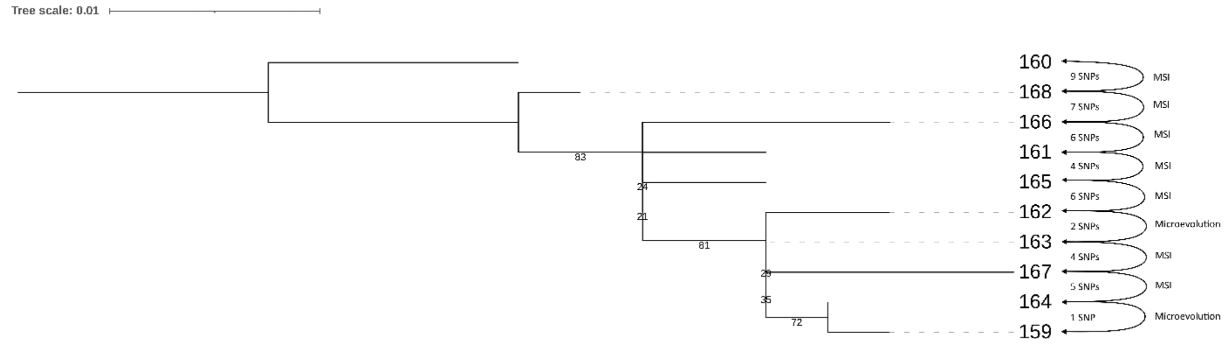

(b) A20 Core SNP Phylogeny (>4 SNPs required for MSI classification)

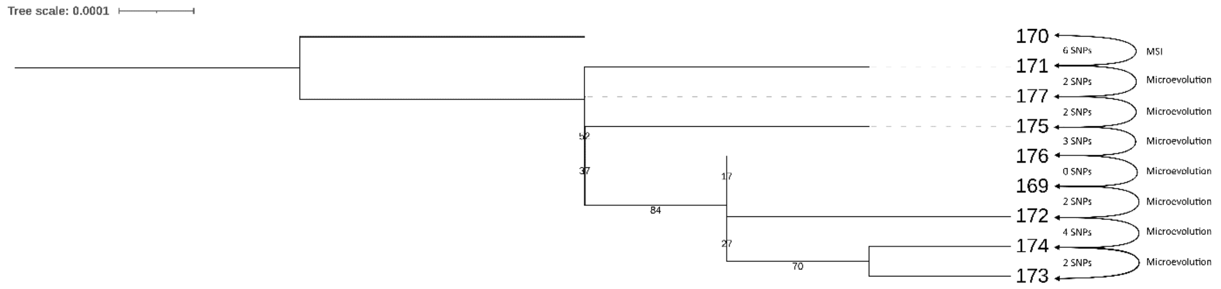

(c) A21 Core SNP Phylogeny (>3 SNPs required for MSI classification)

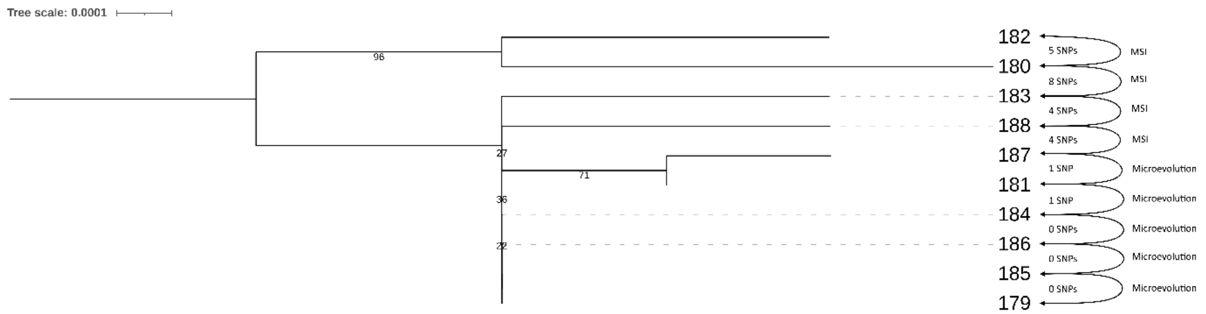

Supplementary Figure S6: (Continued)

(d) A22 Core SNP Phylogeny (>6 SNPs required for MSI classification)

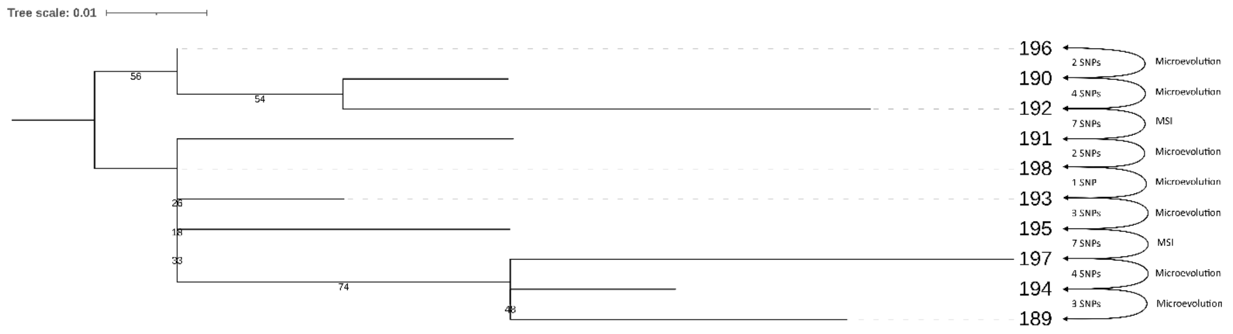

(e) A23 Core SNP Phylogeny (>4 SNPs required for MSI classification)

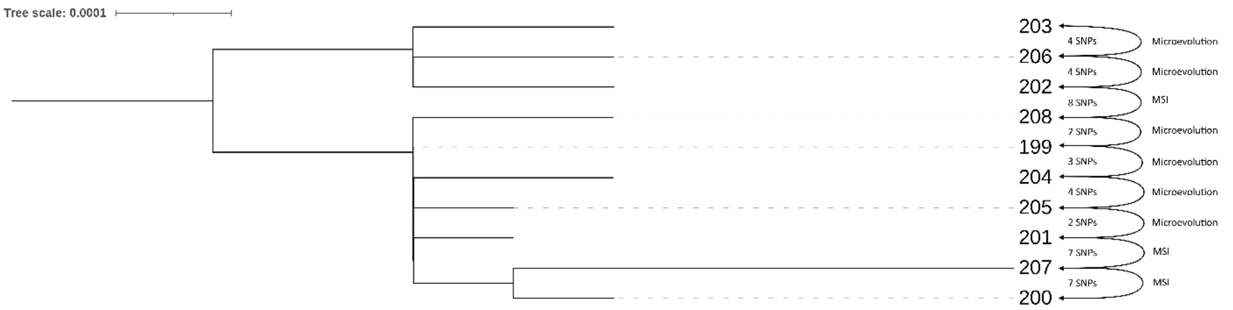

(f) A26 Core SNP Phylogeny (>3 SNPs required for MSI classification)

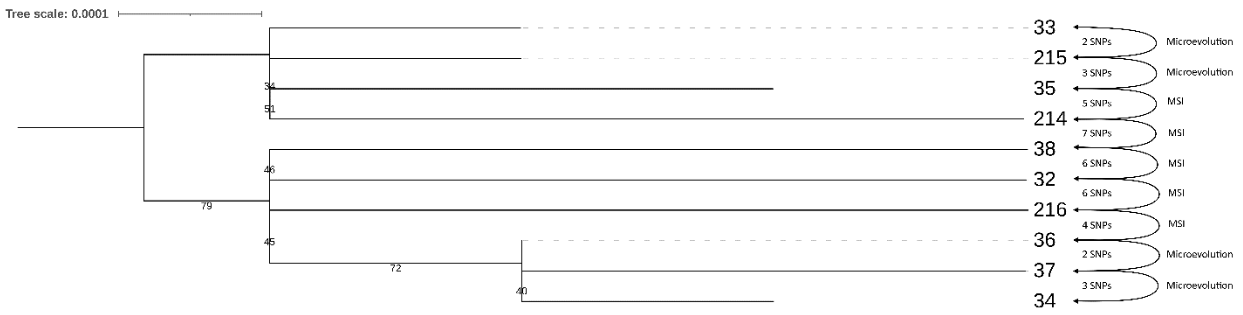

Supplementary Figure S6: (Continued)

(g) A37 Core SNP Phylogeny (>3 SNPs required for MSI classification)

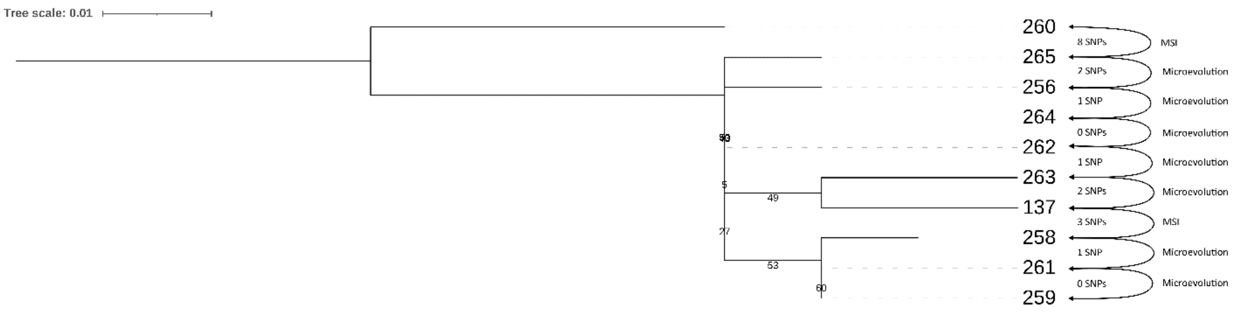

(h) AM1 Core SNP Phylogeny (>3 SNPs required for MSI classification)

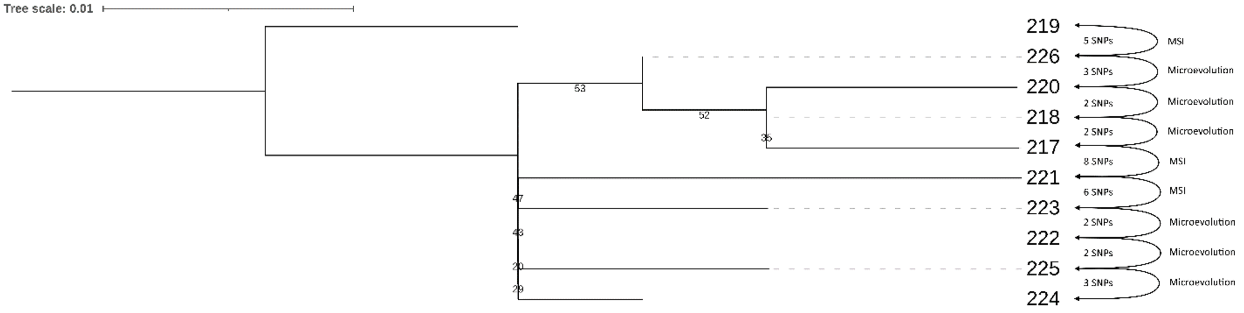

(i) AM1 Core SNP Phylogeny (>3 SNPs required for MSI classification)

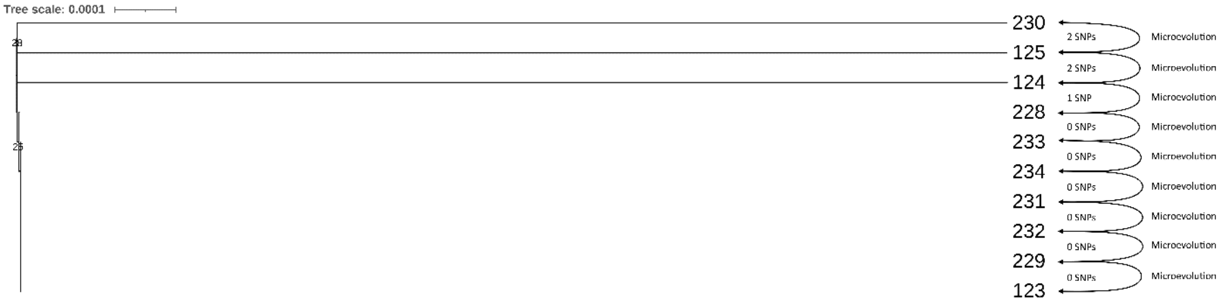

Supplementary Figure S7: Presence of Sec secretion signal in ORF-1 as predicted by SignalP 6.0. Results obtained using the amino acid sequence of reading frame 1 (RF-1) are shown, which indicates that the signal peptide is cleaved by Signal Peptidase II. The letters above the protein sequence and in the caption are indicative of the N-terminal region (N), the center hydrophobic region (H), and the conserved cysteine in the +1 cleavage site (CS) of lipoproteins (c) found within the signal peptide. Regions labelled “O” are not part of the signal peptide sequence.

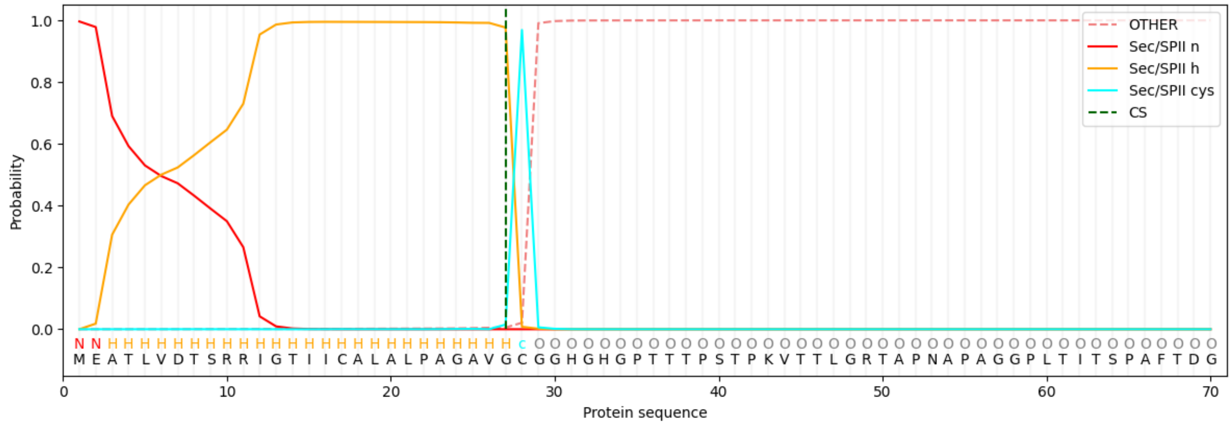

References:

Byrne, A., Ollier, S., Tahlan, K., Biet, F., & Bissonnette, N. (2023). Genomic epidemiology of Mycobacterium avium subsp. paratuberculosis isolates from Canadian dairy herds provides evidence for multiple infection events. *Frontiers in Genetics*, *14*, 110. https://doi.org/10.3389/FGENE.2023.1043598.
